# Fluorescent tagging of VP22 in N-terminus reveals that VP22 favors Marek’s disease virus (MDV) virulence in chickens and allows morphogenesis study in MD tumor cells

**DOI:** 10.1186/1297-9716-44-125

**Published:** 2013-12-21

**Authors:** Sylvie Rémy, Caroline Blondeau, Yves Le Vern, Monique Lemesle, Jean-François Vautherot, Caroline Denesvre

**Affiliations:** 1 INRA, UMR1282, Infectious Diseases and Public Health, ISP, BIOVA team, F-37380 Nouzilly, France; 2 Present address: Medical Research Council Centre for Medical Molecular Virology, Division of Infection and Immunity, University College London, London, UK; 3 INRA, UMR1282, Infectious Diseases and Public Health, ISP, Cytometry facility, F-37380 Nouzilly, France; 4 Département des Microscopies (Plateau Technologique Analyse des systèmes Biologiques), Université François Rabelais, Tours, France

## Abstract

Marek’s disease virus (MDV) is an alpha-herpesvirus causing Marek’s disease in chickens, mostly associated with T-cell lymphoma. VP22 is a tegument protein abundantly expressed in cells during the lytic cycle, which is essential for MDV spread in culture. Our aim was to generate a pathogenic MDV expressing a green fluorescent protein (EGFP) fused to the N-terminus of VP22 to better decipher the role of VP22 in vivo and monitor MDV morphogenesis in tumors cells. In culture, rRB-1B EGFP22 led to 1.6-fold smaller plaques than the parental virus. In chickens, the rRB-1B EGFP22 virus was impaired in its ability to induce lymphoma and to spread in contact birds. The MDV genome copy number in blood and feathers during the time course of infection indicated that rRB-1B EGFP22 reached its two major target cells, but had a growth defect in these two tissues. Therefore, the integrity of VP22 is critical for an efficient replication in vivo, for tumor formation and horizontal transmission. An examination of EGFP fluorescence in rRB-1B EGFP22-induced tumors showed that about 0.1% of the cells were in lytic phase. EGFP-positive tumor cells were selected by cytometry and analyzed for MDV morphogenesis by transmission electron microscopy. Only few particles were present per cell, and all types of virions (except mature enveloped virions) were detected unequivocally inside tumor lymphoid cells. These results indicate that MDV morphogenesis in tumor cells is more similar to the morphorgenesis in fibroblastic cells in culture, albeit poorly efficient, than in feather follicle epithelial cells.

## Introduction

Marek’s disease virus (MDV), also referred to as *Gallid herpesvirus 2*, is the causative agent of Marek’s disease (MD) in chicken, a multifaceted disease most widely recognized by the induction of a rapid and extensive malignant T-cell lymphoma. MDV is a type-species of the *Mardivirus* genus (Marek’s disease-like viruses) within the *Alphaherpesvirinae* subfamily of the *Herpesviridae* family. The actual MD physiopathology model was originally proposed by Calnek (reviewed in [1,2]). Upon entry via the respiratory tract associated with the inhalation of infectious dusts or danders, MDV first replicates in B lymphocytes and subsequently in activated T lymphocytes, leading to acute cytolysis. About 7 days post-infection (dpi), the virus enters a latent state in a subset of CD4+ T cells, which may become transformed leading to lymphoma lesions and mortality, with high rates in genetically susceptible animals (90-100%). Tumors are predominantly located in visceral organs, but also in muscles and skin. Early after infection, the virus is presumably transported by infected lymphocytes to the skin, where it replicates in feather follicles epithelium (FFE) and is shed into the environment [3]. Viral genomes are usually detectable by quantitative PCR (qPCR) in blood cells and feather tips in the first week post-infection at 4–7 dpi with virulent and vaccinal strains, and reach higher levels after 10–21 dpi [4-7].

For more than forty years, it has been recognized that MD tumors are a source of infectious MDV when inoculated into recipient chickens. However, MDV particles have rarely been detected by electron microscopy in this tissue (reviewed in [8]); when found, MDV particles were only in a very low number of cells from lymphoblastoid or epithelial origin [9-12]. In these studies, mostly kidney and gonad tumors were analyzed. It is also noticeable that in lymphoblastoid cells from tumors, MDV particles were only observed in the nucleus as naked nucleocapsids or in the perinuclear region as primary-enveloped virions. In such cells, MDV virions were never observed in the cytoplasm as expected in the double envelopment morphogenesis model [13-15]). In that model, the assembly process begins in the nucleus where the viral genome is packaged into capsids, resulting in type C capsids. Then, nucleocapsids exit the nucleus, by budding into the inner membrane of the nuclear envelope as primary-enveloped virions. Next, these virions fuse with the nuclear outer membrane, resulting in the release of capsids in the cytoplasm. Finally, the cytosolic capsids bind several tegument proteins and are re-enveloped by budding into cytoplasmic vesicles, resulting in mature virions, which exit from the cell, probably by exocytosis.

The VP22 protein encoded by UL49 gene is specific to alpha-herpesviruses. This 249 to 304 amino acid protein is a major constituent of the virus tegument layer. In culture, UL49 functional requirements vary by type of alpha-herpesvirus and by host cell. The UL49 gene has been shown to be absolutely necessary for the replication of MDV and VZV [16-18] whereas UL49 is dispensable for Pseudorabies virus (PRV), Herpes Simplex type 1 (HSV1), and Bovine Herpes virus type 1 (BoHV1) [19-22]. In BoHV1, the deletion of UL49 reduced extracellular virus titers of about 10-fold [23] and plaques size in MDBK by 52% [21]. In HSV-1, the absence of UL49 impaired virus growth in MDBK, but not in Vero cells [20]. In vivo, UL49 was found to play a role in the virulence of BoHV1 in cattle and HSV1 in mice, [22,24,25], but was not involved in the virulence of PRV in rodents [19]. We have previously shown that an attenuated recombinant MDV (Bac20) expressing a EGFP fused in the N-terminus (N-term) of VP22 had a 3-fold decrease in plaques size in cell culture [26]. A recombinant MDV expressing a EGFP fused in the C-terminus (C-term) of VP22 in the very virulent RB-1B pathogenic background was recently reported to be highly attenuated, inducing tumors in only 10% of injected chickens [27]. Herein, we constructed a new EGFP-UL49 recombinant MDV in the RB-1B pathogenic background, in which the fluorescent tag was fused in 5′ of the UL49 gene, and investigated its phenotype in susceptible chickens in order to better characterize the role of VP22 in MD pathogenesis. We showed an attenuation in tumor formation by 1.5 to 3-fold, horizontal transmission and virus replication in vivo. Electron microscopic examination of MD tumors expressing EGFP protein showed that these cells have a lymphoid morphology and are producing MDV particles, including rare cytoplasmic ones.

## Materials and methods

### Cells and bacmids

Chicken embryonic skin cells (CESCs) were prepared and cultivated as previously described [28] from 12-day chicken embryos (LD1 Brown Leghorn chicken line). The RB-1B bacmid used in this study corresponds to the repaired Bac RB-1B 1272 [6]. This bacmid was kindly provided by Dr K. Osterrieder.

### Generation of the recombinant rRB-1B EGFP22 bacmid and virus expressing the MDV VP22 fused to EGFP in N-term

**Figure 1:**
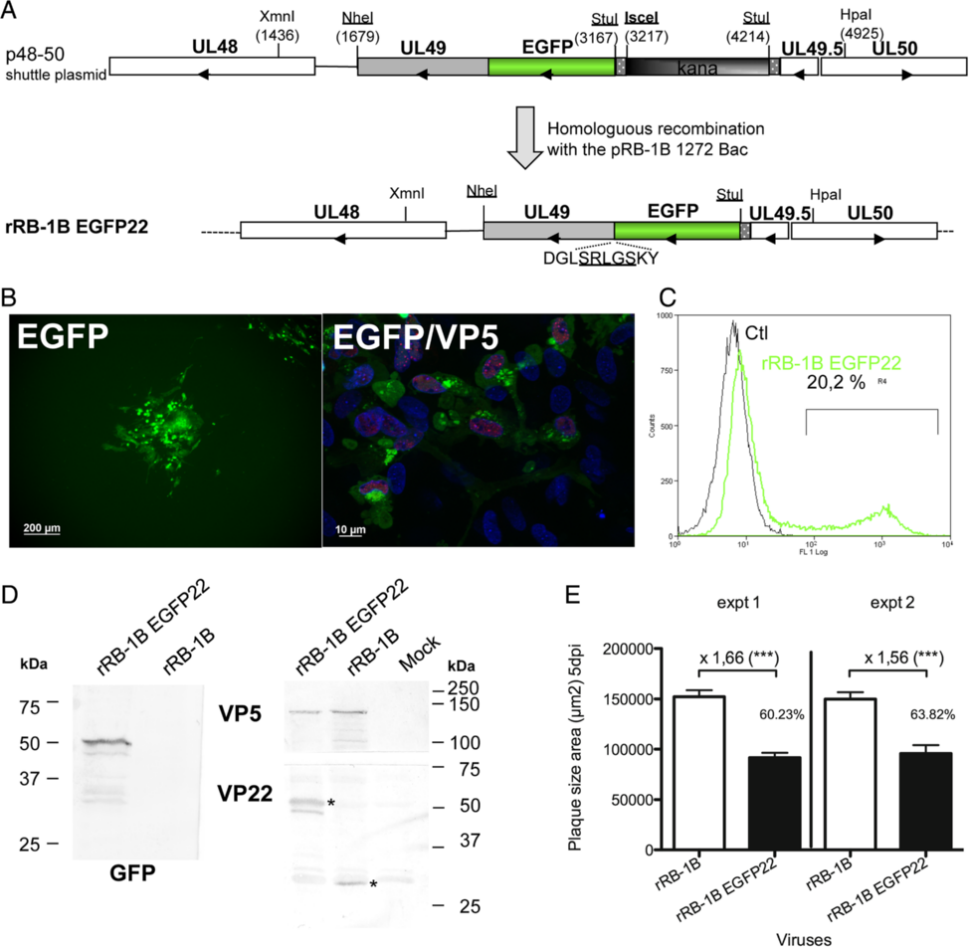
**Construction and characterization of rRB-1B EGFP22 in cell culture. A**. Schematic representation of the shuttle plasmid constructed to generate the rRB-1B EGFP22 mutant by using homologous recombination in *E. coli* with the pRB-1B 1272 DNA bacmid. The shuttle plasmid was derived from the p48-50 StuNhe EGFPUL49 plasmid previously described. **B**. Analysis of EGFP expression by fluorescence. Picture of an infection plaque visualized with EGFP fluorescence at 5 dpi (low magnification). At high magnification, infected cells were stained with an anti-VP5 capsid mouse MAb (red) and Hoechst 33342 dye, staining nuclei (blue). **C**. Flow cytometry analysis on CESCs infected with rRB-1B EGFP22 (green curve) or non-infected (black curve), based on EGFP fluorescence. In this experiment, about 20% of the rRB-1B EGFP22 infected cells were EGFP-positive. **D**. Analysis of EGFPVP22 protein expression in infected cells by immunoblot revealed with an anti-GFP or an anti-MDV VP22 antibody. The stars indicate either the EGFPVP22 or the VP22. An anti-VP5 antibody was used as a control. Mock corresponds to non-infected cells. **E**. Plaques size comparison. At 5 dpi, 50 plaques were stained with a cocktail of anti-MDV antibodies followed by a secondary antibody coupled to Alexafluor 594. Plaques were photographed with the cell observer system (Zeiss, Göttingen, Germany) on the red channel, and measured using the Axiovision software. The error bars represent the standard error of the mean (SEM) of the size of 50 plaques; the values on the graph indicate the plaque size ratio between rRB-1B and rRB-1B EGFP22 (***, *P* < 0.0001).

The Bac RB-1B EGFP22 was generated by “en passant mutagenesis” method [29]. Briefly, for the first recombination step, we used the p48-50 shuttle plasmid schematically represented in Figure 1A, resulting from the insertion in StuI of the Stu-ISceIKana-Stu cassette (1047 bp) into the p48-50 StuNhe EGFPUL49 (previously described in [26,30]). In that last plasmid, the UL48-50 region originated from the RB-1B strain. The first recombination was obtained after transformation of GS1783 bacteria containing the RB-1B 1272 bacmid with the 3489-bp XmnI/HpaI restriction fragment from the shuttle plasmid and kanamycin selection. The second recombination was obtained after inducing the I-SceI expression in order to excise the kanamycin-resistance cassette. After the second recombination step, the mutant bacmid was verified by sequencing the entire region between HpaI and XmnI restriction sites (2442 bp) at 5′ and 3′ ends of EGFPUL49, in which the two recombinations occurred.

The generation of the rRB-1B EGFP22 virus was obtained as follows: 4 × 10^6^ CESCs were transfected with 6 μg of the mutant bacmid by using the calcium phosphate method. Six days later, the cell monolayer showing fluorescent viral plaques was harvested and the virus was amplified by replication on fresh CESCs. In this study, the virus used never exceeded 4 passages in CESCs.

### Detection of VP22 proteins expression by immunoblotting

VP22 was detected in Western-blot either by using an anti-GFP antibody or the L13a MDV VP22 specific antibody as previously described [26] with a few modifications listed below: The infected or non-infected cell monolayers were trypsinized, pelleted, and resuspended in lysis buffer (50 mM NaCl, 0.5 mM DTT, 0.5 mM MgCl2, 2.5 mM Tris HCl pH 8, 0.5% NP40 supplemented with benzonase (25 UI/mL) (Novagen-EMD Biosciences, Billerica, Mass, USA) and protease inhibitors (complete mini EDTA-free, Roche Applied science, Penzberg, Germany) and incubated 1 h 30 at 4 °C. The lysate was centrifugated 30 min at 4 °C at 15 600 *g*. The NP40 insoluble fraction was resuspended in 30 μL of 2 × Laemmli sample buffer and boiled. Solubilized proteins were separated by SDS-PAGE. For the rabbit polyclonal anti-GFP antibody (Clontech, Mountain view, CA, USA), the staining was performed as previously described [26]. For the L13a anti-VP22 mouse monoclonal antibody (MAb), the incubations were performed in Tris NaCl pH 8.25 instead of Tris NaCl pH 7.5.

### Virus cell-to-cell spread by plaques size measurement assay

CESCs (1.5 × 10^6^) grown on 6-well plates were infected with 100 plaque-forming units (pfu) of recombinant rRB-1B 1272 (parental) or rRB-1B EGFP22 (mutant) viruses. At 5 dpi, cell monolayers were fixed with 4% paraformaldehyde (PFA) and plaques were stained with a cocktail of three monoclonal antibodies as previously described [31]. The plaques were observed with a Fluar × 5 objective mounted on an Axiovert 200 M inverted epifluorescence microscope (Zeiss, Göttingen, Germany), photographed with a CCD camera, and measured and analyzed as previously described [32].

### In vivo experiments

Specific pathogen-free White Leghorn chicks (B13/B13 haplotype) were housed in isolation units. Chicks were inoculated intramuscularly (pectoral muscles) with 1000 (experiment 1) or 1500 pfu (experiment 2) of each virus (rRB-1B EGFP22 mutant or rRB-1B parental) at 1 week of age. Birds were evaluated daily for MD symptoms, euthanized, and necropsied when they presented clinical evidence of MD. At the end of the experiments, all surviving birds had their blood sampled, and were euthanized and necropsied. In experiment 1, 12 inoculated birds were housed with 9 or 11 naive birds (contacts) from the beginning of the experiment in order to monitor MDV spread into contacts. Injected and contact surviving birds were euthanized at 90 and between 112–130 dpi, respectively. In experiment 2, 12 inoculated chicks per group were housed in order to measure the viral load in blood and feather tips during the course of the infection. Infected surviving birds were euthanized at 105 dpi. Blood samples (50 μL) from all birds were collected in sodium citrate 75 mM (vol:vol) before infection and at 7, 14, 21, 28, 36 dpi. In addition, 8 axillary tract feathers were collected as described [33] from 10 birds before infection, and at 13, 27, 35 dpi on all birds. After 36 dpi, blood and feather samples were collected only from surviving birds in the rRB-1B EGFP22 group. In addition, blood (5 mL) was collected from the 7 surviving birds in the rRB-1B EGFP22 group at the end of the experiment (105 dpi) for peripheral blood mononuclear cells (PBMCs) preparation. PBMCs were prepared as follows: 10 mL of chicken blood (diluted at 1:2 in phosphate buffer solution [PBS]) was loaded over 5 mL of MSL (medium for lymphocytes isolation, Eurobio, Les Ulis, France). After centrifugation at 2200 rpm for 20 min, PBMCs were collected at the interface plasma/MSL, rinsed twice in PBS, and used for virus isolation on CESCs.

All experimental procedures were conducted with good animal practice and approved by the appropriate local ethic committee ("Comité Régional d'Ethique pour l'Expérimentation Animale", CREEA, protocol number #CL207-40).

### DNA extraction from whole blood and feathers tips

Thirty microliters of blood-citrate was mixed with 1 mL of a cold permeabilizing solution (10% saccharose (w/v), 10 mM Tris HCl 7.5, 5 mM MgCl_2_, 1% Triton X-100) and immediately centrifuged 5 min at 1100 *g* to remove hemoglobin. The pellet was next resuspended in 500 μL of lysis buffer (10 mM Tris HCl pH 8, 10 mM NaCl, 10 mM EDTA, and 1 mg/mL proteinase K) and digested overnight at 56 °C. After an extraction with phenol-chloroform, DNA was precipitated with ethanol. Final DNA was eluted in 25 to 100 μL of ultrapure water supplemented with RNAse A at 10 μg/mL (Sigma, St Louis, MO, USA).

For each animal, the pulp and the epithelium of the collected feather tips was extracted mechanically on a small piece of Whatman paper. All samples were harvested individually, except at time 0 at which extractions were performed in two pools. For DNA extraction, the Whatman paper was soaked in 500 μL of lysis buffer (10 mM Tris HCl pH 8, 0.5% SDS, 0.5 mg/mL proteinase K) overnight at 56 °C. The following steps of the DNA extraction were performed as for the blood (described above).

### Quantification of MDV genome copies by qPCR

Quantification of MDV genome copies using qPCR was performed using the TaqMan technology, as previously described by Jarosinski et al. [6,34]. Primers and probes sequences (reported in [34]) were obtained from Eurogentec. The iNos and the ICP4 probes were tagged with FAM-BHQ1 and Yakima Yellow-BHQ1, respectively. Each qPCR mixture contained 10 μL of 2 × Fast Blue qPCR master mix (Eurogentec, Seraing, Belgium), 9.5 μL of diluted DNA, 10 pmol of each gene-specific primer, 5 pmol of the gene-specific probe in a 20 μL volume. ICP4 and iNos genes were quantified independently on triplicates. The standard curve for ICP4 was obtained by performing qPCR on a serial 10-fold dilution of a bacmid containing the entire MDV genome (Bac20) starting at 4.75 ng (23.1 × 10^6^ copies). The standard curve for iNos was performed in the same manner, starting from 475 pg (56.8 × 10^6^ copies) of a pBS iNos plasmid. The positive cut-off points correspond to ≥ 23 and 57 copies of viral DNA and iNos, according to the standard curves. All qPCR were performed in a Dyad Disciple chromo 4 apparatus (BioRad, Marnes-la-Coquette, France) and the results were analyzed using the MJ opticon monitor software (version 3.1) (BioRad). For each sample, the number of MDV genome copies per 10^6^ cells was calculated based on the number of ICP4 copies per 10^6^ iNos copies.

### Sorting of EGFP-positive cells by flow cytometry from MD tumors developed by rRB-1B EGFP22-infected chickens

Tumors from different organs (gonads, kidneys, or spleen) were collected freshly after chicken death into a large volume of RPMI medium, cut into 1-cm^3^ pieces, rinsed twice in PBS, and crushed over a 100-μm filter. After mechanical dissociation, tumor cell suspension was resuspended into 20 mL of PBS and overlaid on 10 mL of MSL (see above). After centrifugation, the cells at the inferface were harvested, rinsed twice in 15 mL of EMEM. An aliquot of each cell preparation was taken for analysis by fluorescence microscopy. Cells were fixed with 4% PFA for 1 h at room temperature, and subsequently mixed with 1 volume of PBS, and stored at 4 °C until cell sorting, 1 to 4 days after fixation. Just before sorting, cells were resuspended and filtered on a 30-μm pore-size membrane. EGFP-positive cells were then sorted with a MoFlo (DakoCytomation A/S, Fort Collins, USA) high-speed cell sorter as previously described [26]. The only difference was the purification mode which was enriched, a mode that does not eliminate doublets of positive and negative cell, and allow the purification of more EGFP-positive cells than with the “purified mode”, but with a lower purity. The enriched mode was chosen because of the low percentage of EGFP-positive cells, in order to not lose positive cells. The sorted cells were collected in 4% PFA for all purposes.

### Fluorescence microscopy

#### ***MDV infected cells in culture***

CESCs grown on coverslips were infected with the rRB-1B EGFP22 or the parental virus with 100 pfu and fixed 5 dpi with 4% PFA. Cells were then stained with an anti-MDV VP5 monoclonal antibody as previously described [26] and observed on an Axiovert 200 M inverted epi-fluorescence microscope equipped with a 40× PlanNeofluar oil/Dic objective or a 63× PlanApochromat oil/DIC, both with the ApoTome system (Zeiss). Images were captured with a CCD Axiocam MRm camera (Zeiss) using the Axiovision software (Zeiss).

#### ***Explanted tumor cells***

EGFP-sorted and non-sorted tumor cells were centrifuged at low speed with a cytospin (Shandon Southern) on a 0.17-μm glass coverslip coated with poly-L-lysine solution (Sigma-Aldrich, St Louis, Mo, USA), fixed and stained with Hoechst 33342 before observation by fluorescence microscopy as described above. Cells from one tumor were stained with an antibody anti-chicken CD4 (clone CT-4, Southern biotech, Birmingham, AL, USA) followed by a donkey anti-mouse IgG Texas red (Jacskon laboratory, Bar Harbor, ME, USA).

### Transmission electron microscopy (TEM)

Approximately 38 000 EGFP-enriched cells derived from a rRB-1B EGFP22-induced tumor (Testis #16) were pelleted and prepared as previously described for TEM [35]. Ultrathin sections (100-nm thick) were cut, placed on EM grids, and stained with 5% uranyl acetate plus 5% lead citrate. All sections were observed either with a Jeol 1011 or a Jeol 1230 microscope (JEOL, Tokyo, Japan) equipped with a ES1000W Erlangshen CDD Camera (Gatan, Pleasanton, Calif.). Images were captured through Digital Micrograph software version 3.11.1 (Gatan).

## Results

### Generation of rRB-1B EGFP22 MDV

We inserted and fused the EGFP sequence in 5′ of the UL49 gene in the context of the repaired pathogenic rRB-1B bacmid, as schematized in Figure 1A. The mutated bacmid, Bac RB-1B EGFP22, was verified by sequencing the complete region between the XmnI/HpaI restriction sites (2442 bp) (Figure 1A). A viral progeny obtained after transfection into CESCs was amplified and its phenotype was analyzed in cell culture. The EGFP signal appeared in cells expressing other late antigens like VP5 capsid protein, indicating a lytic infection (Figure 1B). The EGFP signal was intense and allowed an easy detection of infected cells by microscopy or cytometry (Figure 1B and C), on live or PFA-fixed cells. The apparent molecular mass of the tagged EGFPVP22 protein appeared as a doublet in Western-blot, with a major form of about 50 kDa (Figure 1D), as previously shown in another MDV genetic background [26]. In addition, the rRB-1B EGFP22 virus showed a reduction in cell-to-cell spread, with a significant 1.5- to 1.7-fold decrease in plaques size compared with the parental virus (as measured in two independent experiments; *P* < 0.0001; Figure 1E).

### Comparison of rRB-1B EGFP22 with rRB-1B parental virus regarding lymphoma formation and horizontal dissemination in chickens

**Figure 2:**
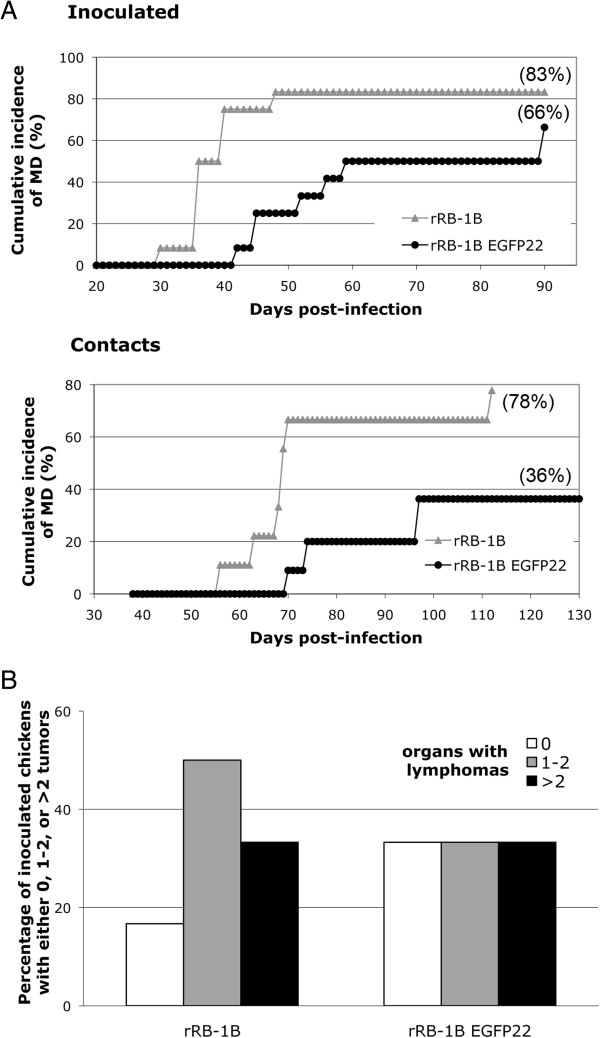
**MD incidence and tumor formation in chickens infected with the rRB-1B EGFP22 virus. A**. Cumulative incidence of MD in birds inoculated with rRB-1B EGFP22 or rRB-1B viruses (*n* = 12), and contact naive birds of the same age (*n* = 9 to 11) housed together (Experiment 1). Viruses (1000 pfu) were inoculated intramuscularly into groups of 1 week-old, B13/B13 White Leghorn chickens. MD incidence was determined by identification of gross lesions at the necropsy. In both groups, at the end of the experiment, a few chickens showing no clinical signs had tumors. The cumulative incidence of the disease is expressed as a percentage. **B**. Lymphoma incidence in inoculated birds of each group, according to the number of organs presenting macroscopic MD lesions in each bird.

In order to evaluate the phenotype of the rRB-1B EGFP22 virus in vivo, 1 week-old MD-susceptible B13/B13 White Leghorn chicks were inoculated intramuscularly with 1000 pfu of the rRB-1B EGFP22 or the parental virus. rRB-1B EGFP22 and rRB-1B induced tumors in 66% and 83% of the inoculated birds (*P* < 0.05; Fisher’s exact test), respectively, with a mean time to disease onset of 59 and 36 dpi, respectively (*P* < 0.05; Mann–Whitney U test) (Figure 2A). Moreover, in both groups 33% of the inoculated birds had tumors in three or more organs, suggesting that tumors-induced by both viruses presented no difference in their ability to spread to multiple organs (defined herein as aggressiveness) (Figure 2B). All together, these results indicated that the rRB-1B EGFP22 virus is partially attenuated in its efficiency to induce lymphoma, but not in its tumor aggressiveness.

The incidence of MD was also evaluated in contact birds (Figure 2A). At the end of the experiment, 78% of the contacts in the rRB-1B group developed tumors while only 36% did in the rRB-1B EGFP22 group. In addition, in the rRB-1B EGFP22 contact group, only 1 out of the 7 surviving birds presented MDV antibody seroconversion, suggesting that most surviving contact birds were not infected (not shown). These results showed that the rRB1B EGFP22 is impaired in bird-to-bird dissemination compared with the rRB-1B virus. This suggests that the mutant virus is either weakly shed from the inoculated birds, less stable in the environment, and/or less infectious by the respiratory route than the parental virus.

### Viral load in blood and feathers of chickens infected with rRB-1B EGFP22 and rRB-1B

In order to explore whether the lower tumorigenicity in inoculated birds and the lower transmission to contact birds of rRB-1B EGFP22 was associated with a decrease in viral replication in lymphocytes and feather follicles, we performed a second in vivo experiment in which we measured MDV genome copy number by real-time qPCR in whole blood and feather tips of injected birds. In this experiment, the chickens received a higher dose of virus inoculum (1500 pfu in Expt 2 vs 1000 pfu in Expt 1) in order to determine whether a higher dose would increase MD incidence in the rRB-1B EGFP group. The rRB-1B EGFP22 and rRB-1B viruses induced tumors in 33% and 100% of the injected birds, respectively confirming that the rRB-1B EGFP22 virus is attenuated in its ability to induce lymphoma. Surprisingly, increasing the rRB-1B EGFP inoculum dose did not increase MD incidence, but had even a contrary effect. The difference in tumor incidence between the two experiments could not be attributed to the inoculum, as the same viral stock was used in both experiments.

**Figure 3:**
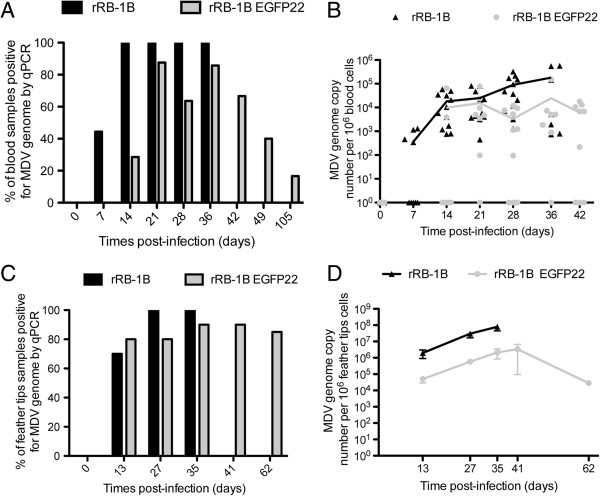
**MDV genome copy number in blood cells and feather tips of chickens.** The rRB-1B EGFP22 or rRB-1B virus (1500 pfu) was inoculated intramuscularly in 1 week-old White Leghorn B13/B13 chickens (Experiment 2). Wing vein blood and axillary tract feathers were collected at different times post-infection. For all samples, DNA was extracted and examined for ICP4 and iNos by qPCR. **A**. % of ICP4-positive chickens after blood examination by qPCR over time. **B**. Viral loads (MDV genome copy number per million cells) in blood per chicken during the course of infection, with means curves for ICP4-positive birds. **C**. % of ICP4-positive chickens after feather tips examination by qPCR over time. **D**. Mean ± SEM viral loads in feather tips per group over time.

DNA samples were prepared from blood or axillary feather tips at various time points in both groups. DNA was extracted individually (except for feathers at time 0) and analyzed for the ICP4 viral gene and iNOS cellular gene by qPCR using the TaqMan technology. MDV genome copy number per million cells was determined by using standard curves for ICP4 and iNOS. In the rRB-1B group, the MDV genome was detectable in the blood of all infected birds examined until 36 dpi, except at 7 dpi (90.3% ICP4 positive samples, *n* = 52) (Figure 3A). Most of viral loads measured were between 10^3^ and 10^6^ genome copies per million blood cells (Figure 3B). The mean of the viral load increased progressively over time to reach 1.8 × 10^5^ at 36 dpi.

In the rRB-1B EGFP22 group, MDV DNA was detectable in 67% of the blood samples analyzed between 14 and 36 dpi (*n* = 33) (Figure 3A). The MDV genome was undetectable in the blood at 7 dpi, suggesting a delay in replication. All birds except one were found positive for the virus at least once. The viral loads measured in the positive blood samples were usually between 10^3^ and 10^5^ viral DNA copies per million cells (Figure 3B). When rRB-1B EGFP22 was detectable, the mean viral load was about 2- to 20-fold lower than in the rRB-1B group (1.8, 1.6, 26.0, and 7.3-fold lower at 14, 21, 28 and 36 dpi, respectively). In addition, rRB-1B EGFP22 mean viral loads did not progressively increase over time as rRB-1B mean viral loads did, suggesting that rRB-1B EGFP22 might be better controlled by the host. All together, these results indicate that rRB-1B EGFP22 is delayed in its replication in blood cells by at least 7 days, and displayed lower viral loads at all time points during the course of infection compared with the rRB-1B.

In the feather tips, the MDV genome was detectable by qPCR in 89.6% and 85.0% of the samples tested for rRB-1B and rRB-1B EGFP22 group, respectively (Figure 3C). At 2 week pi, 80% of the rRB-1B EGFP22-injected chickens were ICP4-positive in feathers whereas only 28% were in blood. The lower detection in blood is compatible with the fact that blood contains a small fraction of infectable cells, lymphocytes being less than 0.5% of nucleated blood cells. In the rRB-1B group, the mean viral load was above 10^6^ genome copies per million cells at all time points. The mean viral loads were 35- to 52-fold lower in the rRB-1B EGFP22 group in this tissue (Figure 3D). These differences were significant at 27 and 35 dpi between the two groups (Mann–Whitney; *P* < 0.001).

Infectious rRB-1B EGFP22 virus was re-isolated from the PBMCs of all surviving birds, after one (6/7 birds) or two passages (1/7 bird) on CESCs in culture (not shown), including in the bird which was always negative by PCR on blood. This result indicates that the rRB-1B EGFP22 genome was present in the PBMCs of all surviving birds, probably in a latent state and able to reactivate in culture.

### A small percentage of EGFPVP22 expressing cells are present and purifiable from rRB-1B EGFP22-induced tumors

**Figure 4:**
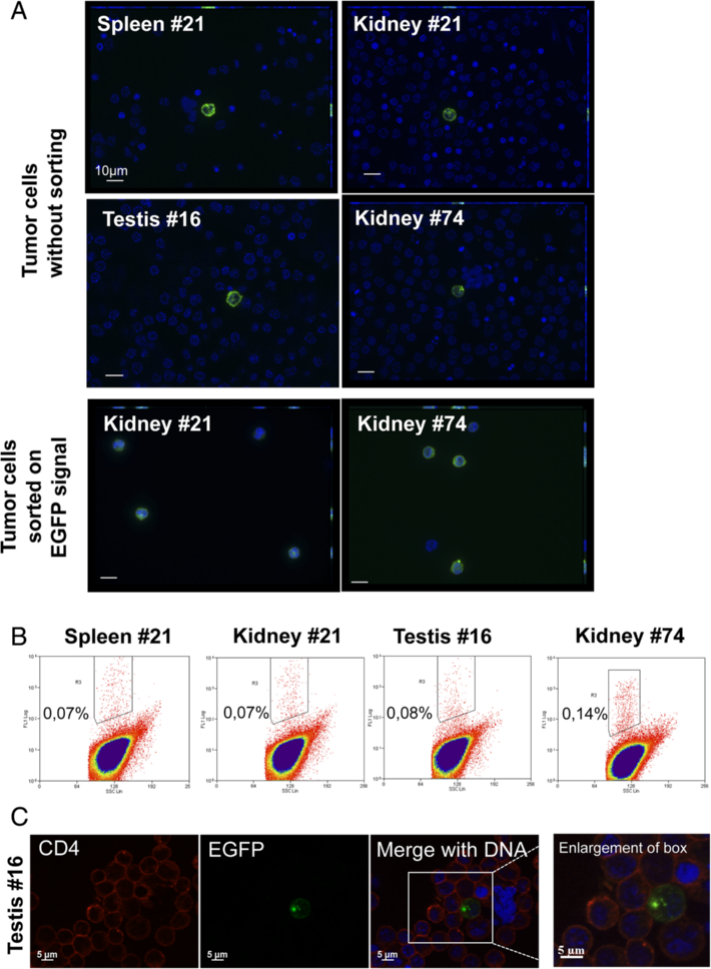
**EGFP fluorescent cells in rRB-1B EGFP22-induced tumors and selection by cell-sorting. A**. Microscopy analysis of tumor cells with or without cell-sorting on the basis of EGFP fluorescence. Tumor cells, purified on MSL, were attached to glass coverslips by cytospin, stained by Hoechst 33342 dye (blue) and analyzed by fluorescence microscopy for EGFP signal and labelled nuclei. Cells from four different tumors are presented without cell-sorting. Cells from kidney #21 and #74 are presented with cell-sorting demonstrating the enrichment in EGFP-positive cells. Bars represent 10 μm. **B**. Flow cytometry analysis of tumor cells from four different rRB-RB EGFP22-induced tumors, after MSL purification. The percentage of EGFP-positive cells in each cell preparation is indicated on each FL1/SSC graph. **C**. Cells from a tumor (testis #16), without cell sorting, examined after CD4 antibody staining for CD4 and EGFP signal.

A fluorescent tag fused to a viral gene is a valuable tool to detect herpesvirus infected cells in tissues, especially in those containing a low number of infected cells [36]. Herein, with the tag fused to a major MDV tegument protein, we could expect to observe a fluorescent signal only in cells which are in lytic phase, either after neoinfection or reactivation from latency. Lymphoid cells from four rRB-1B EGFP22-induced tumors originating from three birds and three organs (kidney, testis and spleen) were isolated on a lymphocytes separation medium cushion and analyzed by fluorescence microscopy and flow cytometry, without any cultivation step. A low number of EGFP-positive cells were easily detectable among the tumor cells by both techniques (Figures 4A and B). In flow cytometry, this percentage was estimated between 0.07% and 0.14% in the four tumors (Figure 4B). Examination of the non-sorted cells from the testis tumor showed that most cells (including the EGFP-positive cell) were CD4-positive, with high or low level (Figure 4C). Sorted-cells from the two kidney tumors were re-examined in fluorescence microscopy in order to verify the purity level (Figure 4A). More than 60% of the cells were EGFP-positive after sorting, showing that this procedure allows an efficient enrichment. In these cells, the EGFP signal was localized mostly in the cytoplasm (Figure 4). Most of the cells had a round shape with a small diameter of 7–8 μm, a high nucleus/cytoplasm ratio and a peripheral ring of cortical actin (not shown), indicating that the cells purified have a morphology compatible with the lymphoid lineage. However the presence of few cells from other lineages could not be totally excluded. These results show that a low proportion of tumor cells expressed EGFPVP22, a marker for the lytic phase and that these cells are purifiable by flow cytometry.

### MDV particles are present in EGFPVP22-enriched cells isolated from tumors

**Figure 5:**
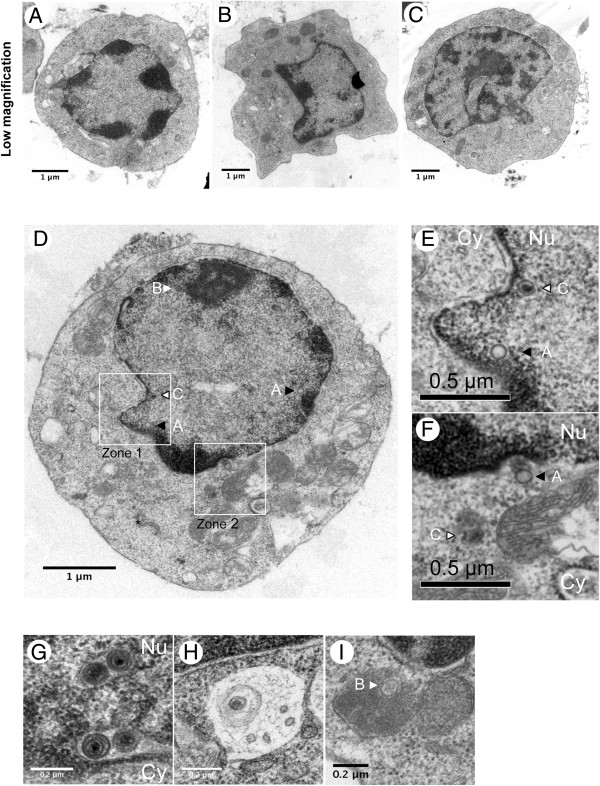
**MDV particles in rRB-1B EGFP22 induced tumor cells examined by TEM.** EGFP-positive cells were enriched from the rRB-1B EGFP22-induced testis tumor (#16). **A**, **B**, **C**. Morphology of sorted-tumor cells presenting herpesvirus particles. **D**. Overview of a cell producing virions, showing different types of particles. **E**. Enlargement of zone 1, drawn in **(D)**, showing two types of naked capsids (type A and C) in the nucleus near the inner nuclear membrane. **F**. Enlargement of zone 2, drawn in **(D)**, showing two naked capsids (type A and C) in the cytoplasm near a mitochondrion. **G**, **H**, **I**. Different types of enveloped or atypical particles. **G**. Vacuole or an invagination in the nucleus containing primary enveloped virions. **H**. A cytoplasmic particle in a vesicle. **I**. Atypical cytoplasmic particle consisting of a B capsid surrounded with a large electron dense material and a membrane. Cy, cytoplasm; Nu, nucleus. Black triangle, type A capsid; white triangle, type B capsid; white triangle with a black outline, type C capsid.

In order to examine whether MDV particles could be detected at an ultrastructural level in rRB-1B EGFP22-induced tumors, 38 000 EGFPVP22-enriched cells from the tumor testis (#16) (Figure 4B) were prepared for TEM. The number of cells per grid was modest, with about 20–25 cells per section. At low magnification, most of the cells had a morphology compatible with an immature lymphocyte (Figures 5A-D), regardless of whether they contained viral particles or not. Rare cells had a morphology reminiscent of an epithelial cell (Figure 5B). When MDV particles were present, the number of particles per cell section was low, under ten (Figure 5D). All types of particles were unequivocally observed at least once over the different cell sections examined, except mature enveloped virions (Figures 5D-I). Nuclear capsids predominated over cytoplasmic capsids. Accumulation of perinuclear virions was only observed once (Figure 5G). Among cytoplasmic naked capsids, we observed type C capsids, but also A and B capsids (Figures 5E-F, I). One cytoplasmic particle possibly enveloped, of about 220 nm in diameter, was observed which was devoid of tegument (Figure 5H). The number of cells presenting particles being inferior to fifty (probably due to the loss of material in the preparation), a quantitative MDV morphogenesis study could not been performed. Overall, these results show that rRB-1B EGFP22-induced tumor cells present MDV particles, including cytoplasmic particles in low numbers.

## Discussion

Our work provides two important findings that contribute to a better understanding of MDV pathogenesis and morphogenesis. First, we have shown that fusing EGFP to the N-term of VP22 in a pathogenic RB-1B background leads to a partially attenuated virus in vivo. This virus provides a basis for understanding the role of VP22 in replication and spread in its natural target cells, as well as in lymphoma formation. It provides an interesting tool for studying the relationship between the level of virus replication and the pathogenesis. Second, we have demonstrated that this virus allows the detection, quantitation and purification of MD tumor cells in lytic cycle, which has never been reported before. We exploited this feature in order to explore MDV morphogenesis in such cells and showed for the first time the presence of cytoplasmic particles supporting a complete morphogenesis process in these cells. Therefore, our work also highlights the utility of MDV virus expressing a bright fluorescent tag to trace lytically infected cells in chicken MD tumors.

Although VP22 is known to be mandatory in MDV replication in cell culture, unlike for most alpha-herpesviruses except VZV [16-18] the reason of VP22 essential role in MDV biology remains unknown. Fusing EGFP tag in N-term of VP22 in the context of RB-1B genome led to a virus showing a spread defect of 1.6-fold in CESCs in culture. This is in accordance with our previous report in the Bac20 genetic background [26], even if in the RB-1B the tagging led to a less pronounced effect. The origin of this attenuation is unclear as EGFPVP22 exhibits a subcellular location similar to non-tagged VP22 after antibody staining (not shown). Although the region of VP22 between amino acids 16 and 37 was found to be necessary for its DNA-binding activity in vitro [28]*,* no data had so far suggested that the N-term had to be free for VP22 function. The GFP tag in itself may have contributed to VP22 defect, as the GFP protein, in fusion or not, has been reported to aggregate at high concentration [37]. Moreover, we cannot totally exclude a cis effect of EGFP insertion on the expression of the genes adjacent to UL49, like UL49.5 which is essential for MDV replication [38].

We observed that the rRB-1B EGFP22 virus induces 33 to 66% of tumors in injected and contact animals. In comparison, the rRB-1B mutant expressing the VP22 fused to EGFP in C-term induced 10% of tumors in injected chickens and none in contact birds [27]. Therefore, the present study confirms that fusing a EGFP tag to the VP22 reduces MDV lymphoma formation. However, this mutant appears less attenuated than the mutant with the tag in C-term, suggesting that the tag position may differentially affect the tumorigenicity. This assumption should be confirmed in the future by evaluating the two mutants side-by-side in chickens.

If VP22 affects tumor incidence, one important question that remains is how VP22 acts on MDV tumorigenesis. In particular, is VP22 directly involved in the tumorigenesis process in T-lymphocytes or is VP22 indirectly involved, due to a lower infectivity or viral replication in T-lymphocytes. Indeed, Calnek proposed that the transformation process is a relatively rare event governed by a set of probabilities, and that the more transformable targets become infected the greater the likelihood that a successful transformation will occur [2]. In the present study, the indirect involvement of VP22 in pathogenesis is supported by the rRB-1B EGFP22 phenotype (a delayed onset of lytic infection and a lower MDV load in the blood), although the alternative hypothesis cannot be totally ruled out.

By monitoring the development of MD tumors in contact chickens, we showed that rRB-1B EGFP22 has a limited horizontal spread in chickens. Moreover, the viral load in the feathers of injected chickens was 1 to 2 log lower in the rRB-1B EGFP22 group compared with the parental group. In this last group, the viral loads in feather tips were in accordance with a previous report [5]. These results indicate that rRB-1B EGFP22 reached the feather follicles and that its skin tropism was not altered, although its replication in feathers is reduced as in blood cells. The growth defect of the rRB-1B EGFP22 virus is therefore general to all cell lineages tested.

We demonstrated that rRB-1B EGFP22 can be employed to quantify and purify tumor cells in lytic cycle directly from tumors. Although it is partially attenuated for MD pathogenesis in chickens, rRB-1B EGFP22 is, to our knowledge, the only fluorescent virus efficient for such purpose. Indeed, with the rRB-1B UL47EGFP virus, which is not attenuated in vivo, the authors failed to detect fluorescent cells from tumors (see Figure six in [27]). rRB-1B EGFP22 yielded a small proportion (0.15%) of the tumors cells expressing EGFPVP22 which is compatible with previous analyses showing that most tumor cells are latently infected, and that none to very few express lytic antigens [9,39]. Futhermore, if EGFPVP22 expression in lymphomas reflects MDV reactivation, the fact that we observed a low percentage of EGFPVP22-positive cells in our study like with wild-type virus suggests that the rRB-1B EGFP22 mutant is not impaired in the reactivation process.

In this report, by coupling the use of a fluorescent MDV mutant with cell-sorting and TEM, we easily observed the presence of MDV particles in a MD testis tumor. This result is remarkable because, since MDV discovery in 1967, most researchers have failed to detect MDV particles in tumors [40-42] or only after extensive study of a high number of tumors [11]. Herein, only one tumor was examined and was found positive for MDV particles, indicating that our approach is technically possible and efficient in order to study MDV morphogenesis in tumors and potentially in other tissues or organs. It is noticeable that the number of particles per cell section observed in tumor cells (between one to ten) was lower than in CESCs infected with Bac20 EGFPVP22 in culture [26]. In our opinion, the low percentage of cells detected during the lytic cycle in tumors and the low number of particles per cell section is sufficient to explain the difficulty to detect MDV virions in MD tumors after direct examination by TEM. It is also remarkable that most cells selected from MD tumors that we observed, with or without particles (not shown), had an ultrastructure comparable to the one previously reported [40].

This report provides the first images of MDV particles in the cytoplasm of lymphoid cells directly from a MD tumor. Even though we did not examine enough cells to perform a reliable quantitative study, cytoplasmic capsids were beyond doubt in minority compared with nuclear particles, as previously observed in cell culture. In addition, the three types of naked capsids (A, B, and C) were visualized in the cytoplasm of cells in which the nuclear envelopes were not disrupted, which is unusual for an alphaherpesvirus in normal conditions [43]. Therefore, one important question that remains unanswered is whether MDV low titers are a consequence of a nuclear egress defect. Taken into consideration that only 0.5% of particles were mature enveloped virions in fibroblastic cells in culture [26], the failure to detect such virions in tumor cells was not totally surprising due to the low number of total particles observed. To reduce animal experiments and have an unlimited cells source, an interesting approach would be to generate and use a lymphoid cell line established from a MD tumor induced by a fluorescent virus, which has no growth defect and expresses its fluorescent tag exclusively during the lytic cycle.

In conclusion, we show that a N-term EGFP tagging of VP22 alters MDV replication *in vivo* in blood cells and feathers as well as lymphoma induction and bird-to-bird transmission showing that VP22 contributes to MDV virulence. Although attenuated, this fluorescent virus allows to select MD tumor cells in lytic cycle. Our work demonstrates for a second time that the combination of fluorescent MDV, recent imaging techniques, and TEM provides new opportunities to examine MDV morphogenesis in various cell context, including harvested from infected birds.

## Abbreviations

CESCs: Chicken embryonic skin cells; C-term: C-terminus; dpi: Days post-infection; EGFP: Enhanced green fluorescent protein; MD: Marek’s disease; MDV: Marek’s disease virus or *Gallid herpesvirus 2*; Mab: Monoclonal antibody; N-term: N-terminus; PBS: Phosphate buffered solution; PFA: Paraformaldehyde.

## Competing interests

The authors declare that they have no competing interests.

## Authors’ contributions

SR carried out all qPCR experiments, participated to the rRB-1B in vitro characterization, and to the animal experiments. CB generated the rRB-1B EGFP22 virus and contributed to its in vitro characterization. YLV performed the cell sorting experiments. ML prepared TEM samples for observation. JFV contributed to animal experiments, the set-up of qPCR, and discussions on the manuscript. CD conceived the study, performed the in vivo experiments, collected and prepared the tumors for cell sorting, carried out all observations by microscopy, analyzed the results and wrote the manuscript. All authors read and approved the final manuscript.
